# An integrated strategy involving high‐throughput sequencing to characterize an unknown GM wheat event in Canada

**DOI:** 10.1111/pbi.14232

**Published:** 2023-12-05

**Authors:** Marie‐Claude Gagnon, Marc‐Olivier Duceppe, Adam Colville, Louise Pope, Marie‐José Côté, Dele Ogunremi

**Affiliations:** ^1^ Canadian Food Inspection Agency (CFIA) Ottawa Ontario Canada

**Keywords:** diagnostic qPCR assay development, characterization unknown GM event, transgenic wheat, high‐throughput sequencing, genome walking

## Abstract

Glyphosate‐resistant wheat plants were discovered in southern Alberta in 2017, representing an unauthorized GM release in Canada. The Canadian Food Inspection Agency undertook a series of experiments to characterize and identify this unknown GM wheat, as well as to develop and validate construct‐specific and event‐specific qPCR assays. Results of PCR‐based assays and Sanger sequencing indicated the presence of CaMV 35S promoter (p35S), Rice Actin 1 intron (RactInt1), CP4‐EPSPS gene and nopaline synthase terminator (tNOS) elements in the unknown GM wheat. Genome walking and bead capture strategies, combined with high‐throughput sequencing, were used to identify the 5′ and 3′ wheat junctions and the subsequent mapping of the insert to chromosome 3B of the wheat genome. A probable transformation vector, pMON25497, was recognized, and further testing identified the unknown GM wheat as MON71200 event, one of two events obtained with the pMON25497 vector. The two construct‐specific assays targeted the junctions of the RactInt1 and the CP4‐EPSPS elements and the CP4‐EPSPS and tNOS elements, while the event‐specific assay was located at the 3′ junction into the wheat genome. Both construct‐specific and event‐specific assays had limits of detection of 0.10% of MON71200 in a seed pool. As expected, the two construct‐specific assays cross‐reacted with other wheat and corn events containing the same elements in the same order. No cross‐reactivity was observed for the event‐specific assay. The integrated strategy employed in this study can serve as a model for other cases when facing similar challenges involving unknown GM events.

## Introduction

During the 2017 growing season, glyphosate‐resistant wheat plants were discovered along an access road in southern Alberta, Canada. Upon notification of this finding by Alberta Agriculture and Forestry, the Canadian Food Inspection Agency (CFIA) undertook a series of risk‐based analyses to determine the origin and potential diffusion of these wheat plants (CFIA, 2018). As genetically modified (GM) wheat is not authorized for commercial production in most countries, the finding of glyphosate‐resistant wheat plants represented an unauthorized GM release in Canada. Unauthorized releases of genetically modified organisms (GMOs) often impact trade and the local economy (US‐GAO, 2008), particularly when they involve crops of high commercial importance such as wheat in Canada. The characterization and identification of this unknown, unauthorized GM wheat event, as well as the development of validated diagnostic tests, were carried out in a timely fashion by CFIA to manage this issue of the utmost importance, both domestically and internationally.

Multiple PCR‐based methods can be used to characterize and identify unknown GM events. These methods can be broadly categorized as (1) element‐specific, (2) construct‐specific and (3) event‐specific methods (Fraiture *et al*., 2015a; Holst‐Jensen *et al*., 2012). Element‐specific methods are mainly used for screening to determine whether a sample contains genetically modified material or not and to provide clues to identify this GM event by revealing which elements are present. Construct‐specific methods target the junction of two or more GM elements that occur together in a GMO. These methods are useful for screening and identifying any GMO possessing a certain genetic construct. However, they are not event‐specific as several GM lines and different crops may have been transformed with the same construct (Fraiture *et al*., 2015a; Holst‐Jensen *et al*., 2012). Finally, event‐specific methods target the junction of the host genome and the vector used for genetic transformation (Holst‐Jensen *et al*., 2012). However, the development of event‐specific methods requires knowledge of the transgene sequence, full or partial, and the sequence of at least one flanking region in the host genome. Because of the cost and time required for identifying the insertion site and developing an event‐specific assay, this work is not usually undertaken for GM lines early in development, but only once an elite line is nearing a stage of commercial release. Therefore, acquiring such knowledge can be challenging, especially in the case of unknown and/or uncharacterized GM events since they do not have to go through an authorization process.

Unknown or partially unknown GM events can be fully characterized using targeted strategies such as genome walking and/or target enrichment combined with high‐throughput sequencing (HTS) and/or Sanger sequencing (Fraiture *et al*., 2017, 2019a). Genome walking, performed with restriction‐based, extension‐based and/or PCR‐based methods, can be used to determine the DNA sequence of unknown genomic regions flanking a known DNA sequence (Fraiture *et al*., 2014, 2015a, 2018b, 2019a; Shapter and Waters, 2014). In the case of partially characterized GMOs, genome walking can be anchored on the known transgenic elements present in the unknown event. For example, the flanking sequences of several suspected GM petunia samples circulating on the EU market have been characterized using a PCR‐based DNA walking method anchored on known transgenic elements combined with MinION sequencing of the resulting PCR products (Fraiture *et al*., 2019b).

Target enrichment, that is the selective capture of DNA strands containing genomic regions of interest through different hybridization techniques, coupled with sequencing can also be used to help characterize partially known GMOs (Mamanova *et al*., 2010). Although target enrichment is frequently used in the HTS of eukaryote genomes, this strategy has not been widely applied to the characterization of GMOs (Fraiture *et al*., 2015a). Debode *et al*. (2019) developed a database of sequences of 10 promoters, 6 terminators and 23 genes commonly found in genetically modified plants. Capture probes were created using the sequences contained in this database and used to enrich DNA libraries of different known GMO samples. These samples were sequenced on an Illumina MiSeq system, and constructs for all GMO samples tested were recovered successfully. In some instances, the technique also allowed the sequencing of the junctions between the plant genome and GM constructs (Debode *et al*., 2019).

Unknown, uncharacterized and potentially unauthorized GM events can be fully characterized using whole‐genome sequencing (WGS) (Fraiture *et al*., 2015a, 2017, 2019a). In this approach, the entire DNA of an unknown GM sample is submitted to HTS. WGS data can be assembled *de novo* or with the help of a reference genome when available through bioinformatics, including the whole transgenic cassette(s) and insertion site(s) characterization (Fraiture *et al*., 2017). The WGS data set can also be used to bait only the reads containing known transgenic elements, therefore reducing significantly the computing requirements for the assembly process (Fraiture *et al*., 2015a). This approach has been successfully used to characterize many GMOs, such as the MON17903 and MON87704 soya bean events, the LLRICE62, TT51‐1 and T1c‐19 rice events and the FP967 flax event (Kovalic *et al*., 2012; Wahler *et al*., 2013; Yang *et al*., 2013; Young *et al*., 2015).

The unauthorized GM wheat event found in Canada was completely unknown and uncharacterized upon its discovery in 2017. To perform trace‐forward/trace‐back activities and design diagnostic assays to share with trading partners, the CFIA initiated a full characterization of this GM wheat event. The objectives of this study were to (i) characterize the genetic elements present in the transgenic cassette(s) of the GM wheat event using PCR and Sanger sequencing; (ii) determine the identity and order of the elements of the full transgenic cassette(s) and the 5′ and 3′ junctions of this event in the wheat genome using genome walking and target enrichment combined with MinION (Oxford Nanopore Technologies) sequencing; and (iii) design and validate a construct‐specific and an event‐specific qPCR assays for the detection and identification of this unknown GM wheat.

## Results

### Sanger sequencing

**Figure 1:**
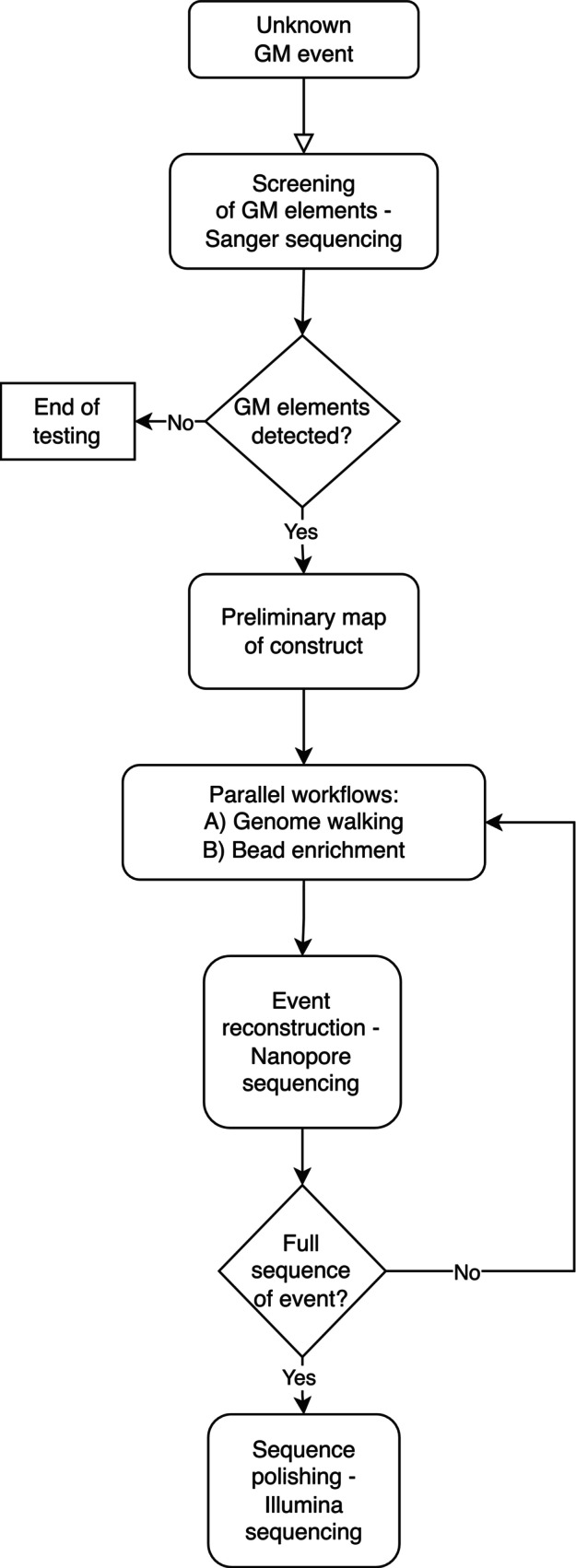
Flow chart of the strategies used to characterize and identify an unknown GM wheat event.

Unknown GM wheat samples were first screened by PCR and Sanger sequencing for the presence of transgenic elements (Figure 1) known to be used in the production of several transgenic wheat lines (BCH, 2023; Euginius, 2023). Amplification products and high‐quality sequences were obtained for p35S (cauliflower mosaic virus (CaMV) 35S promoter), RactInt1 (Rice Actin 1 intron), CP4‐EPSPS (*Agrobacterium tumefaciens* 5‐enolpyruvylshikimate‐3‐phosphate synthase) and tNOS (*A. tumefaciens* nopaline synthase terminator) elements (Lipp *et al*., 2001; Matsuoka *et al*., 2002), revealing their presence in the unknown GM wheat (Table 1). The PCR assay (Matsuoka *et al*., 2002) used for the detection of the pRact1 (Rice Actin 1 promoter) did not yield any detectable amplification product, suggesting its absence from the unknown GM wheat (Table 1).

**Table 1:** Primers and PCR conditions of transgenic elements and element combinations amplified and/or Sanger sequenced in order to characterize an unknown GM wheat event in Canada

Element (s)	Forward primer	Reverse primer	PCR product	dNTPs (mm)	Primer [μm]	Taq units (U)	Annealing T (°C)	PCR cycles
CP4‐EPSPS	ALLRR‐1F^†^	ALLRR‐2R^†^	YES	0.80	1.0	0.50	64	35
p35S	p35S‐F^‡^	p35S‐R^‡^	YES	0.64	0.2	1.00	62	50
tNOS	tNOS‐F^‡^	tNOS‐R^‡^	YES	0.80	0.6	1.00	62	50
pRact1	rACT pro 2–5′^§^	rACT pro 1‐3′^§^	NO	0.80	0.2	1.25	63; 60	9; 29
RactInt1	RactInt1‐1F^†^	RactInt1‐1R^†^	YES	0.80	1.0	1.00	64	30
RactInt1/CP4‐EPSPS	RactInt1‐1F^†^	ALLRR‐2R^†^	YES	0.80	0.5	1.25	64	40
CP4‐EPSPS/tNOS	ALLRR‐1F^†^	tNOS‐R^‡^	YES	0.80	0.5	1.25	62	40
RactInt1/tNOS	RactInt1‐1F^†^	tNOS‐R^‡^	YES	0.80	0.5	1.25	62	40
p35S/RactInt1	p35S‐F^‡^	RactInt1‐1R^†^	YES	0.80	0.5	1.25	62	40
p35S/CP4‐EPSPS	p35S‐F^‡^	ALLRR‐2R^†^	YES	0.80	0.5	1.25	62	40
p35S/tNOS	p35S‐F^‡^	tNOS‐R^‡^	NO	0.80	0.5	1.25	64	40
tNOS/p35S	tNOS‐F^‡^	p35S‐R^‡^	YES	0.80	0.5	1.25	62	50
tNOS/RactInt1	tNOS‐F^‡^	RactInt1‐1R^†^	YES	0.80	0.5	1.25	62	50
pRact1/p35S	rACT pro 1–3′^§^	p35S‐R^‡^	YES	0.20	0.8	0.05	55	35
pRact1/RactInt1	rACT pro 1–3′^§^	RactInt1‐1R^†^	YES	0.20	0.8	0.05	55	35
pRact1 RC/p35S	rACTpro1‐3RevComp^†^	p35S‐R^‡^	YES	0.20	0.8	0.05	55	35
pRact1 RC/RactInt1	rACTpro1‐3RevComp^†^	RactInt1‐1R^†^	YES	0.20	0.8	0.05	55	35
pRact1 RC/CP4‐EPSPS	rACTpro1‐3RevComp^†^	ALLRR‐2R^†^	YES	0.20	0.8	0.05	55	35
p35S/CP4‐EPSPS	p35S‐R^‡^	ALLRR‐2R^†^	YES	0.20	0.8	0.05	55	35
CP4‐EPSPS/p35S	ALLRR‐1F^†^	p35S‐R^‡^	YES	0.80	0.5	1.25	62	50

RC, Reverse Complement.

^†^ In‐house primer.

^‡^ Lipp *et al*. (2001) Eur Food Res Technol 212: 497–504.

^§^ Matsuoka *et al*. (2002) J Agric Food Chem 50: 2100–2109.

Amplification products and high‐quality sequences were also obtained for the combination of forward and reverse primers of all transgenic elements identified in the construct, except for the combination of p35S forward primer with tNOS reverse primer (Table 1). The amplification products obtained for each primer combination provided information on the orientation (e.g. 5′ to 3′) and the order of sequence of each transgenic element (e.g. p35S, followed by RactInt1, followed by CP4‐EPSPS and then by tNOS), while the number of PCR products obtained for each combination of primers provided information on the number of copies of each element in the construct (e.g. at least two copies of each element). This information was used to draft a preliminary map of the construct.

### High‐throughput sequencing

Five rounds of genome walking were performed using primers of transgenic elements identified previously (p35S, RactInt1, CP4‐EPSPS and tNOS) combined with pools of degenerated primers to characterize the neighbouring genetic elements and determine the transgene insertion site in the wheat genome (Table 2). All primers from the transgenic elements present in the construct generated amplification products suitable for HTS when combined with the degenerated primers pools of the genome walking kit. However, the CP4‐EPSPS primer was excluded after the initial genome walking round because amplicons generated with this primer dominated the sequence reads obtained (data not shown).

**Table 2:** Genome walks and MinION runs completed to characterize an unknown GM wheat event in Canada

Genome walk #	Element‐specific primer	Degenerate primer pool	MinION kit	Trimmed reads	Baited reads	Mapped reads (of baited)	Mapped reads from total reads	Mapped on chloroplast
1	tNOS‐F^†^	A	SQK‐LSK108 (Ligation Sequencing Kit)	1 445 622	16 474	7247 (43.99%)	7247 (0.50%)	54 (0.00%)
	p35S‐R^†^	B						
	ALLRR‐1F^‡^	C						
	RactInt1‐1R^‡^	D						
2	tNOS‐F^†^	A^§^	SQK‐RBK004 (Rapid Barcoding Kit)	174 341	865	230 (26.44%)	230 (0.13%)	233 (0.13%)
	p35S‐R^†^	B^§^						
	ALLRR‐1F^‡^	C^§^						
	RactInt1‐1R^‡^	D^§^						
	tNOS‐F^†^	D						
	tNOS‐R^†^	A						
	p35S‐R^†^	B						
	p35S‐F^†^	C						
3	rACTpro1‐3RevComp^‡^	A, B, C, D	SQK‐LSK108 (Ligation Sequencing Kit)	617 577	7815	6 (0.08%)	6 (0.001%)	65 (0.01%)
4	p35S‐R^†^	A, B, C, D	SQK‐LSK108 (Ligation Sequencing Kit)	170 216	1903	16 (0.84%)	16 (0.01%)	43 (0.03%)
5	RactInt1‐1R^‡^	A, B, C, D	SQK‐LSK108 (Ligation Sequencing Kit)	207 865	9273	3074 (33.15%)	3074 (1.48%)	65 (0.03%)
Total	―	―	―	2 615 621	36 330	10 573 (29.10%)	10 573 (0.40%)	460 (0.02%)

^†^ Lipp *et al*. (2001) Eur Food Res Technol 212: 497–504.

^‡^ In‐house primer.

^§^ From genome walk #1.

Amplification products of the genome walking experiments were submitted to sequencing by MinION, producing over 2.6 M reads in total. Only 1.4% of those reads were baited using the high‐quality Sanger sequences produced as reference. Further filtering of the reads by mapping resulted in a final yield of 0.4%. The map‐filtered reads were used to improve the genetic map of the unknown wheat event previously created from Sanger sequencing data. This process was iterative, and multiple combinations of baiting sequences were used. As the genetic map of the insertion was expanding, specific directions and regions were targeted to increase sequence read depth as needed to uncover the inserted DNA sequence and the transgene junction site sequences (Figure 1). A few reads from the genome walking run with the p35S‐R primer span over almost all the insert length (data not shown).

**Figure 2:**
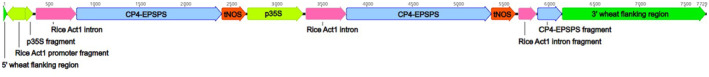
Genetic map of the MON71200 transgene reconstructed from Sanger and high‐throughput sequencing data.

The bead capture/target enrichment strategy was pursued in parallel to the genome walking strategy to identify elements and sequences at the 5′ and 3′ junctions of the transgene (Figure 1). More than 16 M MinION reads (16806428) were sequenced, from which only 129 037 (0.77%) were baited and 43 784 (0.26%) remained after mapping and filtering (Table 3). Long reads from the selective enrichment allowed investigators to extend the sequence in the host genome and increase overall confidence in the genetic map of the transgene (Figure 2).

**Table 3:** MinION runs completed with PCR‐labelled biotin probes to characterize an unknown GM wheat event in Canada

MinION run	Trimmed reads	Baited reads	Mapped reads (of baited)	Mapped reads from total reads	Mapped on chloroplast
1	57 271	6	0	0	0
2	72 303	7	0	0	0
3	189 099	26	0	0	0
4	437 511	61 701	42 857 (69.46%)	42 857 (9.80%)	808 (0.18%)
5	1 768 917	10 828	1 (0.01%)	1 (0.00%)	4581 (0.26%)
6	7 514 128	33 658	554 (1.65%)	554 (0.01%)	28 255 (0.38%)
7	11 935	56	1 (1.79%)	1 (0.01%)	27 (0.21%)
8	1 413 498	4700	74 (1.57%)	74 (0.01%)	3943 (0.28%)
9	5 341 766	18 055	295 (1.63%)	297 (0.01%)	12 105 (0.23%)
Total	16 806 428	129 037	43 782 (33.93%)	43 784 (0.26%)	49 719 (0.30%)

The combination of genome walking and bead capture/target enrichment strategies enabled the identification of both 5′ and 3′ flanking regions into the wheat genome and the location of the insert into chromosome 3B of that genome. The insertion point was located in a low‐complexity region of the genome. Only about 60 bp could be identified with confidence at the 5′ wheat junction because of the presence of highly repeated motifs. On the other hand, 1582 bp of the 3′ wheat junction was identified. The 3′ flanking region was repeated multiple times in the wheat genome, and most of the baited reads not mapping to the final event sequence had high similarity to parts of the 3′ wheat junction sequence. The transgene contained two copies of the p35S/RactInt1/CP4‐EPSPS/tNOS cassette, although the first one has a truncated p35S combined with a partial copy of the pRact1 (Figure 2). No T‐DNA was detected, nor were any antibiotic resistance genes (or any other selection markers). The presence of more than one cassette, their direct orientation with truncated and some inverted elements, and the absence of T‐DNA borders indicated that the transformation was performed by biolistic bombardment rather than with *Agrobacterium*.

Because nanopore sequencing is prone to errors (the average error rate of this study was around 10%), a functional EPSPS protein sequence could not be obtained from the MinION sequence data. To resolve this issue, a final run of Illumina MiSeq was conducted (Figure 1). Paired‐end reads allowed the investigation to polish the sequence of the insert and to predict functional CP4‐EPSPS proteins. The complete sequence of the event has been published in GenBank under the accession number MN020371.

A transformation vector (pMON25497 from Monsanto) was identified as the source of the insert based on the presence of the different transgenic elements detected, their order and a transformation by biolistic bombardment (Hu *et al*., 2003). Reference material and two event‐specific PCR protocols (for 25 372 and 25 397 events) were later received by CFIA from Monsanto to detect the presence of pMON25497 in the unknown wheat. This construct is present in both Monsanto event 25 372 and in MON71200 (also known as event 25 397). Results from these two PCR protocols indicated that the unknown wheat was negative for event 25 372 but positive for the presence of the MON71200 event. *Acc‐1* wheat reference gene positive and negative controls worked as expected (data not shown).

Significant efforts were invested in sequencing the 5′ wheat flanking region, but only 60 base pairs were recovered. The 5′ junction seemed to harbour extremely low‐complexity DNA containing sequence repeats and homopolymers. It was hypothesized that the limited success to sequence the 5′ wheat flanking region could be due to the presence of a secondary structure incompatible with the different molecular biology and HTS technologies used. A genome walking run targeting the 5′ junction was also sequenced using a third HTS technology, the Ion Torrent S5, without more success (data not shown).

The 3′ wheat flanking sequence could also be considered to be located in a low‐complexity region. A BLAST search of the reverse primer of the MON71200 event‐specific assay on the Chinese Spring wheat genome (accession number GCA_002220415.2) returned over 50 priming sites (data not shown). This is not surprising as previous studies have shown that the wheat genome is composed of about 80% transposable element‐derived sequences, the large chromosome 3B being no exception (Choulet *et al*., 2010; Daron *et al*., 2014; Paux *et al*., 2006; Smith and Flavell, 1975).

### Optimization of diagnostic assays

CFIA's construct‐specific qPCR assay was designed around two targets for increased specificity. Optimization trials for these assays showed that concentrations of 10 and 100 pg of DNA of MON71200 were not consistently detected when the TaqMan™ Universal PCR Master Mix was used, with primer/probe concentrations of either 500/100 nm or 400/200 nm and annealing temperatures of 56 or 60 °C. The Luna® Universal Probe qPCR Master Mix, on the other hand, lowered Ct values for all DNA concentrations tested, allowing the detection of 10 (0.01%) and 100 (0.10%) pg of DNA of MON71200 consistently for both tests. Primer/probe concentrations of 400/200 nm and an annealing temperature of 57 °C further improved qPCR amplification for both construct‐specific assays (Table 4).

**Table 4:** Primers and probes for construct‐specific and event‐specific qPCR assays developed for an unknown GM wheat event

Type	Name	Primer/probe sequence	Amplicon size (bp)	Annealing T (°C)	LOD (% in 100 ng total wheat)
Construct qPCR assay 1	RINTEpsps‐1F	TGACAAATGCAGCCTCGT	83	57	0.1
	RintronEPSPS‐1R	ACACCATTGCAGATTCTGC			
	RINTEPSPS‐P	5HEX/TGTAGGTAG/ZEN/AAGTGATCAACCATGGCGCAAGT/3IABkFQ			
Construct qPCR assay 2	EPSPSNos‐1F	GATCGAACTCTCCGATACGA	90	57	0.1
	EpspsNOS‐1R	GGATTCAATCTTAAGAAACTTTATTG			
	EPSPSNOS‐P	56‐FAM/CCTGATGAG/ZEN/CTCGAATTCCCGATCGTTCAA/3IABkFQ			
Event qPCR assay	MON71200‐3′Junction‐1F	CACGACGGTCATCGAGC	139	Touchdown 60 to 55	0.1
	MON71200‐3′Junction‐1R	CCGTTCGTCATTGACTGTT			
	MON71200‐3′Junction‐P	5HEX/CATACGGAA/ZEN/AAGATGCTGCAGGGAATATATTGAAC/3IABkFQ			
Endogene qPCR assay	Wheat acc F (SQ50542)^†^	GCCTACCCCCTTCAACAAGA	94	Both 57 and Touchdown 60 to 55	NA
	Wheat acc R (SQ50543)^†^	ATGTACGCGCTTGAACCCTT			
	Wheat acc P (PB50161)^†^	6FAM/CCACCGACG/ZEN/AGTTAAAACCAAAGATACACG/3IABkFQ			

LOD, Limit of detection.

^†^ European Commission JRC 2016, MON71800 Report.

CFIA's event‐specific qPCR assay was designed at the 3′ junction of the MON71200 event into the wheat genome. Optimization trials for this assay were performed with the Luna® Universal Probe qPCR Master Mix, as this master mix had performed better than the TaqMan™ Universal PCR Master Mix in the optimization of the two construct‐specific qPCR assays. Rounds of optimization performed at 57 °C showed that doubling the concentration of the probe (from 200 nm to 400 nm) did not improve the performance of the assay but that increasing the concentration of the reverse primer to 800 nm improved consistency in the detection of 100 pg of MON71200. Changing the qPCR conditions to a touchdown procedure from 60 to 55 °C further improved the detection power for this assay (Table 4). Further tests with the TaqMan™ Universal PCR Master Mix and the TaqMan™ GTXpress qPCR Master Mix showed that these master mixes worked under the optimized conditions for the event‐specific assay (touchdown procedure and primer/probe concentrations of 400/800/200) but yielded higher Ct values than the Luna® Universal Probe qPCR Master Mix (data not shown).

### Limit of detection and cross‐reactivity

For the two construct‐specific qPCR assays and for the event‐specific qPCR assay, the empirical limit of detection determined on seed pools was 0.10% of MON71200 (Table 5). In fact, all tests gave inconsistent Ct values for concentrations of MON71200 below that level (exp. 0.03% and 0.01%) and, as expected, were negative for the four samples of non‐GM wheat tested (Table 5).

**Table 5:** Validation data and limit of detection of construct‐specific and event‐specific qPCR assays developed for an unknown GM wheat event

Sample name	% GM	Construct‐specific qPCR assay 1		Construct‐specific qPCR assay 2		Event‐specific qPCR assay		Endogenous qPCR assay	
		Ct mean^†^	Ct SD^‡^	Ct mean^†^	Ct SD^‡^	Ct mean^†^	Ct SD^‡^	Ct mean^†^	Ct SD^‡^
BIO‐rMIRL3‐17‐975‐01A	2.00	29.36	0.31	29.35	0.05	25.56	0.12	21.85	0.53
BIO‐rMIRL3‐17‐975‐02A	2.00	28.98	0.24	29.34	0.11	26.82	0.05	23.50	0.21
BIO‐rMIRL3‐17‐980‐01A	2.00	29.51	0.01	29.62	0.10	26.76	0.20	22.74	0.49
BIO‐rMIRL3‐17‐980‐02A	2.00	29.51	0.15	29.35	0.10	27.12	0.26	22.98	0.37
BIO‐rMIRL3‐17‐976‐01A	1.00	29.98	0.28	30.10	0.34	26.75	0.18	21.90	0.80
BIO‐rMIRL3‐17‐976‐02A^§^	1.00	33.52	0.79	32.84	0.41	30.02	0.48	25.29	0.15
BIO‐rMIRL3‐17‐981‐01A	1.00	30.34	0.14	30.61	0.20	26.85	0.16	22.15	0.47
BIO‐rMIRL3‐17‐981‐02A	1.00	30.07	0.19	29.90	0.13	27.30	0.06	22.52	0.53
BIO‐rMIRL3‐17‐977‐01A	0.10	33.58	0.19	34.26	0.79	29.96	0.08	22.49	0.71
BIO‐rMIRL3‐17‐977‐02A	0.10	33.53	0.48	33.93	0.64	30.15	0.93	21.92	0.61
BIO‐rMIRL3‐17‐982‐01A	0.10	33.62	0.04	34.43	0.42	30.65	1.03	22.18	0.47
BIO‐rMIRL3‐17‐982‐02A	0.10	34.17	0.85	34.14	0.22	29.56	0.81	21.79	0.54
BIO‐rMIRL3‐17‐978‐01A	0.03	35.33	1.51	35.01	0.61	33.96	3.32	23.11	0.21
BIO‐rMIRL3‐17‐978‐02A	0.03	34.90	0.63	35.05	0.69	32.31	2.99	22.05	0.74
BIO‐rMIRL3‐17‐983‐01A	0.03	37.89	NA	36.20	NA	31.54	0.96	22.12	0.34
BIO‐rMIRL3‐17‐983‐02A^§^	0.03	37.12	NA	**―**	NA	**―**	NA	25.16	0.10
BIO‐rMIRL3‐17‐979‐01A	0.01	**―**	NA	36.72	NA	32.03	NA	22.72	0.50
BIO‐rMIRL3‐17‐979‐02A	0.01	**―**	NA	**―**	NA	32.28	NA	21.65	0.75
BIO‐rMIRL3‐17‐984‐01A	0.01	33.81	0.81	33.29	0.19	29.97	0.45	22.85	0.23
BIO‐rMIRL3‐17‐984‐02A	0.01	**―**	NA	**―**	NA	―	NA	23.22	0.56
BIO‐rMIRL3‐17‐973‐06A	0.00	**―**	NA	**―**	NA	**―**	NA	22.71	0.97
BIO‐rMIRL3‐17‐973‐07A	0.00	**―**	NA	**―**	NA	**―**	NA	24.32	0.50
BIO‐rMIRL3‐17‐974‐06A	0.00	**―**	NA	**―**	NA	**―**	NA	24.34	0.39
BIO‐rMIRL3‐17‐974‐07A	0.00	**―**	NA	**―**	NA	**―**	NA	24.81	0.67

^†^ Ct mean calculated from values obtained from 3 repetitions of the assay.

^‡^ Missing (NA) values come from the obtention of undetermined Ct values from two or more repetitions.

^§^ Epoch quantification indicated the presence of RNA in these samples, explaining the higher Ct values obtained.

For all spiking levels tested, both construct‐specific assays yielded Ct values higher than the event‐specific assay; in particular, at 0.10% level Ct values were around 33–34 for the formers and 30 for the latter. This could be due to the repetition of the Rice Actin 1 intron and the CP4‐EPSPS elements within the insert that could have decreased the availability of primers for the reaction. Moreover, it should be mentioned that for the event‐specific assay the cycling parameters had to be optimized leading to a touchdown qPCR protocol, which could have proved more efficient for amplification.

For both construct‐specific qPCR assays, cross‐reactivity was observed with the other wheat events (MON71700 and MON71800) and with two (NK603 and MON88017) of the four corn events, but not with the canola and soya bean events tested (data not shown). On the contrary, no cross‐reactivity was observed for the event‐specific qPCR assay with any of the other events tested (data not shown).

## Discussion

Sanger sequencing and targeted HTS strategies allowed the reconstruction and characterization of an unauthorized GM wheat release in Canada without *a priori* knowledge of the event. Further testing led to the identification of the unknown GM wheat as the MON71200 event, a glyphosate‐resistant wheat line developed by Monsanto. These methods also allowed the determination of the 5′ and 3′ wheat junctions of this event, thus enabling the design of construct‐specific and event‐specific qPCR diagnostic assays.

The main HTS sequencing method chosen by CFIA to characterize the unknown GM wheat event was the MinION sequencing device from Oxford Nanopore because it offers several advantages compared to other HTS sequencing platforms (Ip *et al*., 2015; Jain *et al*., 2016). One of the most interesting characteristics of the MinION is the capacity to sequence fragments of different sizes, from 200 bp to above 2.23 Mbp (Ip *et al*., 2015; Jain *et al*., 2018; Payne *et al*., 2019). This feature was used to sequence all the amplicons generated by the genome walking and bead enrichment targeted strategies without size selection or fragmentation. Not only did this simplify the library preparation procedures but it also facilitated the bioinformatics reconstruction of the event by yielding long reads. Other interesting characteristics of the MinION for this project were that it is a small and portable device (Ip *et al*., 2015; Jain *et al*., 2016); data are available in real time (Loose *et al*., 2016); library preparation and workflows are simple; and the device is relatively affordable (Fraiture *et al*., 2018a). As the reconstruction of the unknown GM wheat was performed under time and resource constraints, these characteristics allowed CFIA to have greater flexibility and responsiveness as workflows could be adapted and regions of interest could be targeted more rapidly depending on the results that were coming out of each run. In regulatory science, accuracy is crucial, but speed is also essential to taking action in a setting of real‐world factors such as shipping, seed purchases and planting. The MinION platform was also used successfully by other enforcement laboratories to characterize different GMOs from plants, including unauthorized ones (Fraiture *et al*., 2018a, 2019b).

Genome walking and bead enrichment were the targeted strategies that were combined with HTS to reconstruct and characterize the unknown GM wheat. To our knowledge, this is the first time that these strategies were combined to reconstruct the whole sequence of an unknown GM event in plants. Genome walking is often used to identify transgene flanking junctions based on a certain knowledge of GM events or constructs. For example, genome walking anchored on different GM elements has been successfully applied to different types of samples containing GMOs, including some unauthorized ones (Fraiture *et al*., 2014, 2015a, 2015b; Spalinskas *et al*., 2013). Although enrichment of DNA libraries with hybridization probes that capture GM elements has been proposed for facilitating the detection and identification of GMOs, this technique has not yet been implemented in routine testing (Debode *et al*., 2019; Fraiture *et al*., 2017). Thus far, both techniques have been used mainly for the detection and identification of known and partially known events, not for reconstructing the whole insert and flanking sequences of an unknown and uncharacterized event, as is the case in this study. The success encountered by combining these strategies (Figure 1), along with the availability of bioinformatics tools and parameters used to process HTS data, should allow other enforcement laboratories to implement this approach when faced with unknown or unauthorized GM events.

Genome walking was chosen because it is a well‐established technique (Fraiture *et al*., 2014, 2015a, 2018b, 2019a; Shapter and Waters, 2014) that can be implemented relatively easily and inexpensively when using PCR‐based kits. The different pools of degenerated primers included in the kit allowed several fragments to be amplified and sequenced at once on the MinION device, contributing to the characterization of the unknown event with data generated from the different targets and increasing the odds of finding informative reads while reducing sequencing costs. Genome walking produced a low abundance of reads spanning over the entire insert, but these reads proved to be of great importance for the characterization of the insert. Genome walking also produced non‐specific chimeric sequence reads, generally longer than 5 kb, that complicated the bioinformatics reconstruction of the unknown wheat event, hence forcing their inspection manually. The non‐specific reads originated from the use of only one round of PCR amplification, rather than the three recommended, to maximize the length of the fragments produced. As some of the nested primers were binding to multiple places due to sequence repeats in the insert and sequence rearrangement during transformation, chimeric and non‐specific reads were produced and not left out during subsequent rounds of nested PCR.

The use of thermal asymmetric interlaced polymerase chain reaction (TAIL‐PCR), a method designed to amplify unknown flanking sites adjacent to known sequences through multiple cycles of PCR performed at different melting temperatures (Singer and Burke, 2003), is a strategy that would not have been as useful as genome walking in the present study. Indeed, precise information on elements adjacent to the unknown flanking sites was scarce prior to the use of genome walking and bead enrichment strategies. This lack of information would not have allowed the design of the three sets of nested insertion‐specific primers required by the TAIL‐PCR method (Singer and Burke, 2003). Moreover, TAIL‐PCR is used in combination with Sanger sequencing, which can be slow to perform if multiple or unspecific PCR products are obtained, and which is also limiting in terms of sequence length produced. In this study, the use of HTS allowed the sequencing of multiple amplicons simultaneously and unlimited sequence lengths to be obtained, which permitted a faster insert sequence discovery process.

To increase the number of relevant reads and decrease the production of chimeric sequences, a bead enrichment strategy was also pursued. Although the bead enrichment allowed an increase in read depth and overall confidence in the genetic map of the unknown wheat event, time and resource constraints prevented improvements to the bead enrichment protocol that would have made it more efficient and successful. For example, blocking oligos specific to repetitive sequences in plants (Fu *et al*., 2010) could have been used and optimized rather than the more general I‐Block casein blocking reagent used in this study. Another example is the use of multiple hybridization rounds before bead capture to increase the proportion of target sequences in the genomic library. Finally, the creation of several capture probes, ahead of time, of genes commonly found in genetically modified plants, would have increased our choices of possible targets and most likely our enrichment efficiency, as demonstrated by Debode *et al*. (2019).

The unknown GM wheat found in Canada has been identified as MON71200 event, a glyphosate‐resistant GM wheat line developed by Monsanto. This event was initially eliminated from the list of potential candidates based on the results from PCR and Sanger sequencing. Indeed, the PCRs using the Rice Actin 1 promoter primers were negative in all our attempts to amplify this region (Table 1). However, processing the HTS data from genome walking and bead capture allowed the recovery of the Rice Actin 1 promoter sequence in the insert, albeit incomplete, in reverse complement and fused with a fragment of p35S (Figure 2), making it clear that the event was MON71200. Other elements differed in the reconstructed map of the MON71200 event compared to the original pMON25497 transgenic cassette (Hu *et al*., 2003). Indeed, additional and incomplete fragments of the Rice Actin 1 intron and the CP4‐EPSPS elements were found at the end of the MON71200 vector, bordering the junction of the event into the 3′ end of the wheat genome. Overall, the presence of more than one cassette, their direct orientation, the absence of T‐DNA borders and chimeric/truncated element near the insertion junctions supported the fact that the transformation of MON71200 was performed by biolistic bombardment rather than with *Agrobacterium* (Hu *et al*., 2003).

In addition to identifying the unknown GM wheat event as MON71200, the whole sequence of the event was used to design construct‐specific and event‐specific qPCR assays. Two construct‐specific assays were designed first as they did not require information from the 5′ or the 3′ junction of the event into the wheat genome. They were designed around two targets (junction Rice Actin 1 intron/CP4‐EPSPS and junction CP4‐EPSPS/tNOS) for increased specificity. As expected, the construct‐specific qPCR assays demonstrated cross‐reactivity with GM wheat events MON71700 and MON71800 (different construct, same succession of GM elements) and with GM corn events NK603 (same construct) and MON88017 (different construct, same succession of GM elements). However, they were immediately useful in CFIA's efforts to determine the diffusion of the GM wheat presence in samples collected from the sides of the access road, nearby fields and grain stored at the site.

Moreover, an event‐specific qPCR assay was designed to enable the specific detection of the MON71200 event. The MON71200 event‐specific assay was designed at the 3′ junction of the insert and chromosome 3B of the wheat genome, as a longer stretch of DNA was available to design primers and a probe. This assay allowed to increase the diagnostic capacity since it did not require running crop‐specific assays in the case of a positive detection in order to rule out the hypothesis of cross‐contamination by events of other species. In addition, the event‐specific test and technical support were offered to trading partners so that they could undertake their own confirmations of the integrity of Canadian wheat shipments.

The integrated approach used in this study to characterize an unknown and unauthorized GM wheat event in Canada allowed its identification as MON71200 and the development of construct‐specific and event‐specific qPCR assays. These tests were used to support diagnostic capacity within Canada, and the event‐specific assay was shared with Canada's trading partners. CFIA has developed a powerful workflow that is now part of its diagnostic toolbox and can serve as an example for other countries facing similar challenges.

## Experimental procedures

### Sampling

Wheat material (seeds, wheat heads, chaff/residual and seedlings) was used for DNA sequencing, assay design and test validation. DNA was extracted from seeds and plant tissue (30–200 mg as starting material) using the DNeasy Plant DNA Mini Kit (QIAGEN, Germantown, MD) following the manufacturer's instructions. A CTAB procedure (Doyle and Doyle, 1990) was also used to extract DNA from chaff/residual material, wheat heads and seedlings using 300–600 mg as starting material. DNA concentrations were measured with an Epoch microplate spectrophotometer (BioTek, Winooski, VT).

### Sanger sequencing

Transgenic elements (Table 1) known to be used in the production of several transgenic wheat lines were assessed by PCR. Amplification products were sequenced to confirm their presence in the unknown GM wheat (Figure 1). Forward and reverse primers of all transgenic elements were also combined, and PCR products, when present, were sequenced (Table 1) to confirm the number of copies of each element, their orientation in the construct and the sequence of each element. High‐quality sequences produced with Sanger sequencing were also used for baiting the HTS data sets (details below).

Amplification reactions were carried out following primer‐specific conditions (Table 1), and amplification products were sequenced in both directions using BigDye Terminator v3.1 Cycle Sequencing Kit (Life Technologies Inc., Carlsbad, CA). Multiple amplification products were extracted and purified using QIAquick Gel Extraction Kit (QIAGEN, Germantown, MD) following the manufacturer's instructions. Sequencing products were run on an ABI 3130 *xl* Genetic Analyzer (Life Technologies Inc., Carlsbad, CA), and consensus sequences were built using BioNumerics v7.6 (Applied Maths NV, Sint‐Martens‐Latem, Belgium).

### High‐throughput sequencing

High‐throughput sequencing (Illumina and Oxford Nanopore Technologies) was used in addition to Sanger sequencing to characterize the unknown GM wheat event and to determine the 5′ and 3′ junctions of this event in the wheat genome (Figure 1). Targeted strategies (DNA genome walking and target enrichment) were used in combination with HTS (Figure 1), as recommended for the characterization of unknown or partially known GMOs (Fraiture *et al*., 2015a, 2018b, 2019a).

Genome walking was first performed using four primers of the previously identified transgenic elements (Table 2) to complete a bidirectional sequencing walk using the APAgene™ GOLD‐RT Kit (Bio S&T, Montréal, QC). The first trial was completed with one round of sequence elongation and amplification with a degenerate primer pool (A–D) followed by Nanopore sequencing of all the elongation products (Genome Walk #1; Table 2). Two additional rounds of genome walking and nanopore sequencing were completed by randomizing the reverse primer (degenerate pool of primers A–D) to vary the elongation products that were obtained for sequencing (Genome Walk #2; Table 2). To increase sequencing depth, specific directions and regions were also targeted (Genome Walks #3–5; Table 2). Library preparation was performed with the SQK‐LSK108 (Ligation Sequencing Kit) or the SQK‐RBK004 (Rapid Barcoding Kit) from Oxford Nanopore Technologies. Sequencing was performed on the MinION using FLO‐MIN‐106 R9.4 flow cells (Oxford Nanopore Technologies, Oxford, UK) and the MinKNOW software v18.01.6 for the full 48‐h run time with no alterations to any voltage scripts.

A bead capture/target enrichment strategy was also used to increase the number of relevant sequence reads and decrease chimeric reads generated by the genome walking strategy (data not shown). An in‐house strategy, modifying a previously published MinION HTS target enrichment protocol (Karamitros and Magiorkinis, 2015), was developed utilizing the MinION and SQK‐LSK108 to hybridize biotin‐labelled DNA molecules to complementary sequences in the unknown GM wheat event to target the 5′ and 3′ junction flanks of the insert (Appendix S1).

Three fragments of 2.5 kb were amplified to cover the whole GM wheat insert, and the PCR products were sequenced on a MiSeq system (Illumina, San Diego, CA). The primers for amplification were chosen based on the limited number of unique priming sites in an attempt to yield approximately equal and single PCR products. The fragments were purified and prepared for HTS using the Nextera DNA Flex Library Preparation Kit and sequenced using MiSeq Nano Flow Cell (500 cycles, V2 chemistry) (Illumina, San Diego, CA).

### Bioinformatic analyses

MinION reads were converted from fast5 to fastq using the Albacore v2.2.7 basecaller (https://github.com/dvera/albacore). Fastq files were demultiplexed and trimmed for sequencing/barcoding adapters using Porechop v0.2.4 (https://github.com/rrwick/Porechop). *In silico* target enrichment (baiting) was done using BBDuk v37.78 from the BBTools suite (https://jgi.doe.gov/data‐and‐tools/bbtools/) using a reference file containing high‐quality Sanger sequences. Various combinations of target sequences were used for the baiting procedure. Baited reads were mapped back to the reference file used for bating with bowtie2 v2.2.8 using the ‘very‐sensitive’ option to reduce non‐insert‐related reads (https://github.com/BenLangmead/bowtie2). Filtered reads were then *de novo* assembled with Canu v1.7 (https://github.com/marbl/canu). Complete list of bioinformatics tools, versions and parameters used to process MinION sequencing data is detailed at https://github.com/adamkoziol/minion_amplicon.

MiSeq reads were cleaned from sequencing adapters and low‐quality bases using BBDuk v37.78. Trimmed reads were used to polish the MinION assembly using Geneious 11.0.5 by aligning both data sets and manually correcting inconsistencies found in the MinION assembly. Final assembly of the event (whole inserted DNA plus 5′ and 3′ wheat flanking regions) was manually annotated based on location and orientation of the transgene constituents, assisted with the Sanger sequencing data (Figure 2).

### Design and optimization of diagnostic assays

The whole sequence of the unknown GM wheat event was used to design primers/probes for a construct‐specific and an event‐specific qPCR assays (Table 4). Assays were optimized by testing different annealing temperatures (56–60 °C), different master mixes (TaqMan™ Universal PCR Master Mix—Life Technologies Inc., Carlsbad, CA and Luna® Universal Probe qPCR Master Mix—New England Biolabs, Ipswich, MA) and different combinations of primer and probe concentrations (500/100 nm, 400/200 nm, 400/400 nm or 400/800/200 nm of primers and probes, respectively) using DNA of one individual unknown GM wheat sample diluted in conventional (non‐GM) wheat in a 1 : 10 serial dilution from 100 ng to 10 pg, one non‐GM wheat sample extracted from bulk seeds and a negative control template. Optimal qPCR conditions were determined based on the consistency of results obtained for the different DNA concentrations tested and their associated Ct values, aiming for the detection of the lowest DNA concentration at the lowest possible Ct value.

The construct‐specific qPCR assays were designed around two targets for increased specificity: the first primer pair/probe was designed at the junction of the Rice Actin Intron and the CP4‐EPSPS elements, and the second primer pair/probe was designed at the junction of the CP4‐EPSPS and NOS terminator elements. The optimized reactions for the two tests were carried out in 20 μL volumes with the following final concentrations: 1X of Luna® Universal Probe qPCR Master Mix (New England Biolabs, Ipswich, MA), 400 nm of forward and reverse primers, 200 nm of probe and 100 ng of template DNA. qPCR conditions were as follows: 1 min at 95 °C, 40 cycles of 15 s at 95 °C and 30 s at 57 °C (including plate reading).

The event‐specific qPCR assay was designed at the 3′ junction of the unknown GM wheat event, and the optimized reactions were carried out in 20 μL volumes with the following final concentrations: 1X of Luna® Universal Probe qPCR Master Mix (New England Biolabs, Ipswich, MA), 1 mm MgCl_2_, 400 nm of forward primer, 800 nm of reverse primer, 200 nm of probe and 100 ng of template DNA. qPCR conditions were as follows: 1 min at 95 °C, 5 cycles of 15 s at 95 °C and 30 s at 60 °C (1 °C decrease per cycle), 40 cycles of 15 s at 95 °C and 30 s at 55 °C (including plate reading).

All qPCRs were run on a ViiA 7 Real‐Time PCR machine (Life Technologies Inc., Carlsbad, CA), and data were analysed with QuantStudio Real‐Time PCR Software v1.2 (Life Technologies Inc., Carlsbad, CA). Wheat endogenous assay was performed for each run done to ensure the integrity of all samples tested.

### Limit of detection and cross‐reactivity

To validate the construct‐specific and event‐specific assays and determine their limit of detection, spiked reference material of the unknown GM wheat event was prepared. Seeds from two conventional wheat varieties (AAC NRG097 and AAC Tenacious) were spiked with seeds from the unknown GM wheat to obtain seed pools of the following GM concentrations: 2%, 1%, 0.1%, 0.03% and 0.01%. Seed pools were prepared based on the number of seeds and were homogenized and ground following an in‐house procedure designed to minimize dust generation and avoid cross‐contamination. DNA was extracted from four samples of 200 mg of ground material for each of the GM concentrations using DNeasy Plant DNA Mini Kit (QIAGEN, Germantown, MD).

For the construct‐specific and event‐specific qPCR assays, the four samples of the different GM concentrations were run in triplicate with their respective optimized qPCR conditions along with one conventional wheat (non‐GM) sample and a negative control template, using 100 ng of DNA as starting material. Mean Ct values and standard deviations were calculated for each of the samples at each of the GM concentrations. The limit of detection was determined as the lowest concentration at which all four samples were detected in each of the three replicates.

To evaluate cross‐reactivity, the construct‐specific and event‐specific qPCR assays were tested against other known GM wheat events supplied by Monsanto (MON71700 and MON71800), as well as against certified reference material for canola (GT73, HCN92/Topas 19/2, MS1‐Rf2, MS8‐Rf3, OXY‐235 and HCN28/T45), corn (MON810, NK603, MON863/810 and MON88017) and soya bean (GTS 40‐3‐2, MON89788, DP356043, DP305423, A2704‐12 and A5547‐127) events. For each of the assays, a sample was deemed to cross‐react if an amplification product was obtained (Ct value different from 0) and all controls displayed expected profiles.

## Accession numbers

GenBank MN020371.

## Conflict of interest

The authors have not declared a conflict of interest.

## Authors contributions

MCG, MOD, AC and MJC designed the experiments. MOD, AC, LP and MJC carried out the experiments. MJC and DO contributed reagents and access to technology. MCG wrote the manuscript with contributions from all the authors. All the authors read and approved the final manuscript.

## Supporting information


**Appendix S1** Bead capture/target enrichment procedure.
