# Supplementary material for: An integrated strategy involving high‐throughput sequencing to characterize an unknown GM wheat event in Canada
Source: Plant Biotechnol J. 2023 Dec 5;22(4):904–14. doi: 10.1111/pbi.14232 (PMC10955494; doi:10.1111/pbi.14232)
Supplement: Supplementary file 1 — Appendix S1 Bead capture/target enrichment procedure. [file PBI-22-904-s001.docx]

**Methods S1**

**Bead capture/target enrichment procedure.**

The genome of an unknown GM wheat sample extracted with a CTAB procedure (Doyle and Doyle 1990) was used as a template to randomly incorporate a nucleotide mix of 25% biotin-11-dUTP (ThermoFisher Scientific, Waltham, MA) and 75% dTTP in PCR amplicons of 200-400 bp for hybridization using HotStar Taq DNA polymerase (QIAGEN, Germantown, MD). The amplification targets for the generation of PCR labeled biotin probes were p35S (Cauliflower mosaic virus (CaMV) 35S promoter), RactInt1 (Rice Actin 1 Intron), CP4-EPSPS (Agrobacterium tumefaciens 5-enolpyruvylshikimate-3-phosphate synthase) and tNOS (*A. tumefaciens* nopaline synthase terminator) elements. Forward and reverse primers of these transgenic elements were also combined and their products used as probes if it yielded a single PCR product.

In parallel to PCR amplification of hybridization probes, ligation sequencing adapters (SQK-LSK108 kit, Oxford Nanopore Technologies) were ligated to a 1 µg pool of the same sample’s DNA sheared to 5 Kb using the Covaris M220 Focused Ultrasonicator (Covaris Inc., Woburn, MA). After AMPure bead cleaning (Beckman-Coulter Inc., Brea, CA), the adapter-ligated DNA pool was eluted in 15 µl of water.

The genomic library was then hybridized against 75 ng of biotinylated probes in 6X saline-sodium citrate (SSC) buffer at 50°C for 90 minutes (denaturation of the probe and 5 Kb fragments for 7 minutes at 95°C, cooled to 50°C with 3% ramp speed, hybridization at 50°C for 90 minutes). The hybridized sequences were captured using streptavidin beads (Promega, Madison, WI**)** that had been blocked in 0.5% I-Block (ThermoFisher Scientific, Waltham, MA) to prevent non-specific interactions. The ssDNA pool of captured target sequences was eluted in 50 µl of water after four rounds of washes in 2X and 1X SSC. The washes were completed according to an in-house protocol (Dr. Guillaume Bilodeau, personal communication).

The ssDNA was then split into four pools and used as the template for PCR to convert it to dsDNA. Amplification reactions were carried out in 50 μl volumes with the following final concentrations: 1X LongAmp^®^ Taq master mix, 0.8 μM of primers Y-TOP **-** 5’-AAT GTACTTCGTTCAGTTACGTATTGC-3’), and a nested reverse primer targeting the region of interest (Table 3), and 10 ng of template DNA. PCR conditions were as follows: 3 min at 95ºC; 15 cycles of 30 s at 95ºC, 30 s at 55°C, and 4.5 min at 65°C; and 10 min at 65ºC.

The PCR reactions were cleaned using AMPure beads (1.8X ratio), quantified by QIAxpert spectrophotometer (QIAGEN, Germantown, MD), and equal amounts combined to run on the MinION following the Ligation Sequencing Kit 1D (SQK-LSK108) protocol.
